# Reduced cardiac volumes in chronic fatigue syndrome associate with plasma volume but not length of disease: a cohort study

**DOI:** 10.1136/openhrt-2015-000381

**Published:** 2016-06-24

**Authors:** Julia L Newton, Andreas Finkelmeyer, George Petrides, James Frith, Tim Hodgson, Laura Maclachlan, Guy MacGowan, Andrew M Blamire

**Affiliations:** 1 Institute of Cellular Medicine, Newcastle upon Tyne, UK; 2 Newcastle University, Newcastle upon Tyne Hospitals NHS Foundation Trust, Newcastle upon Tyne, UK; 3 Newcastle Magnetic Resonance Centre, Newcastle upon Tyne, UK

**Keywords:** AUTONOMIC NERVOUS SYSTEM, QUALITY OF CARE AND OUTCOMES

## Abstract

**Objectives:**

To explore potential mechanisms that underpin the cardiac abnormalities seen in chronic fatigue syndrome (CFS) using non-invasive cardiac impedance, red cell mass and plasma volume measurements.

**Methods:**

Cardiac MR (MR) examinations were performed using 3 T Philips Intera Achieva scanner (Best, NL) in participants with CFS (Fukuda; n=47) and matched case-by-case controls. Total volume (TV), red cell volume (RCV) and plasma volume (PV) measurements were performed (41 CFS and 10 controls) using the indicator dilution technique using simultaneous 51-chromium labelling of red blood cells and 125-iodine labelling of serum albumin.

**Results:**

The CFS group length of history (mean±SD) was 14±10 years. Patients with CFS had significantly reduced end-systolic and end-diastolic volumes together with reduced end-diastolic wall masses (all p<0.0001). Mean±SD RCV was 1565±443 mL with 26/41 (63%) having values below 95% of expected. PV was 2659±529 mL with 13/41 (32%) <95% expected. There were strong positive correlations between TV, RCV and PV and cardiac end-diastolic wall mass (all p<0.0001; r^2^=0.5). Increasing fatigue severity correlated negatively with lower PV (p=0.04; r^2^=0.2). There were no relationships between any MR or volume measurements and length of history, suggesting that deconditioning was unlikely to be the cause of these abnormalities.

**Conclusions:**

This study confirms an association between reduced cardiac volumes and blood volume in CFS. Lack of relationship between length of disease, cardiac and plasma volumes suggests findings are not secondary to deconditioning. The relationship between plasma volume and severity of fatigue symptoms suggests a potential therapeutic target in CFS.

**Key questions**

**What is already known about this subject?**

- Chronic fatigue syndrome (CFS) has been shown to be associated with a range of cardiac abnormalities.
- Studies, to date, have suggested that these abnormalities probably arise because of deconditioning.

**What does this study add?**

- This study has confirmed in a large cohort that there are reductions in cardiac volume in CFS measured using cardiac MRI.
- The degree of these end-diastolic and end-systolic volume abnormalities associates with blood volume.
- The abnormalities seen are not arising secondary to deconditioning.
- Reductions in plasma volume associate with fatigue severity.

**How might this impact on clinical practice?**

- This study reinforces, using state-of-the art MRI, previous findings that there is a cardiac abnormality in those with CFS.
- The finding of hypovolaemia in association with cardiac structural abnormalities and fatigue severity represents a potential therapeutic target.

## Introduction

Studies performed using a range of assessment modalities have shown that chronic fatigue syndrome (CFS) is associated with abnormalities of cardiac function.^1–5^ Echocardiographic and impedance studies have confirmed impaired cardiac contractility^1^ ^2^ and reduced left ventricular (LV) function. Structural cardiac MR has shown reduced end-diastolic dimensions and cardiac output with MR spectroscopy detecting impaired cardiac bioenergetic function.^3^ ^4^ The severity of these cardiac abnormalities also appears to relate to symptom severity.^1^ ^3^ This has led to the suggestion that those with CFS have a primary cardiac abnormality that accounts for at least some of their symptoms. These symptoms include orthostatic intolerance which limits the patient's functional capacity and predicts their quality of life.^5^ Indeed, the small cardiac size shown in previous studies has been shown to be more markedly manifested in patients with CFS with orthostatic intolerance.^6^

Studies have hypothesised that these numerous cardiac abnormalities identified in patients with CFS may occur as a result of hypovolaemia and/or deconditioning.^7^ ^8^ Cardiac studies have led to the description of CFS as a ‘small heart syndrome’^9^ ^10^ described in studies as likely to be due to deconditioning arising in the context of hypovolaemia, rather than a primary cardiac abnormality, the initiator being reduced preload secondary to impaired hydration.

In the current study, we extend our previous cardiac MR studies into a second cohort to confirm our original findings of reduced cardiac volumes, and we go onto explore the potential mechanisms that underpin the cardiac abnormalities, including non-invasive cardiac impedance, and red cell mass and plasma volume (PV) measurements performed in the same individuals.

## Methods

### Participants

Participants were recruited as part of an observational study aimed at understanding the pathogenesis of autonomic dysfunction in patients with CFS. Participants fulfilled the diagnostic criteria for CFS.^11^ The study was approved by the research ethics committee (REC 12/NE/0146, CLRN ID 97805). Participants were not selected positively or negatively according to any criteria other than the fact that they were attending a clinical service and had a Fukuda diagnosis of CFS,^12^ although they were excluded if they screened positive for a major depressive episode as assessed using the Structured Clinical Interview for the Diagnostic and Statistical Manual of Mental Disorders (version IV; SCID-IV)^13^ or were taking vasoactive medication or known to have diabetes. Fatigue impact was assessed by the Fatigue Impact Scale (FIS).^14^

Controls were recruited via notices provided in the hospital and University together with a distribution of posters via the local patient support group where we invited relatives of those with CFS to participate. Controls fulfilled the same inclusion and exclusion criteria as participants with CFS; they were not positively or negatively recruited according to fatigue severity or the presence or absence of particular symptoms. All participants provided written informed consent.

### Procedure

After completing the symptom assessment tools, the Task Force Monitor (CNSystems, Graz, Austria) was applied and participants rested supine in a quiet room with standard temperature and lighting. All measurements were performed at the same time of the day and after a light breakfast. After 10 min unrecorded rest, measurement of heart rate and blood pressure was recorded over 10 min supine rest.

### Cardiac MR

Cardiac examinations were performed using a 3 T Philips Intera Achieva scanner (Best, NL). A dedicated six-channel cardiac coil (Philips, Best, NL) is used with the participants in a supine position and ECG gating (Philips vectorcardiogram, VCG system). Cardiac MR cine imaging is acquired to assess cardiac morphology, and systolic and diastolic function. A stack of balanced steady-state free precession images were obtained in the short-axis view during breath holding covering the entire left ventricle (field of view=350 mm, repetition time/echo time=3.7/1.9 ms, turbo factor 17, flip angle 40°, slice thickness 8 mm, 0 mm gap, 14 slices, 25 phases, resolution 1.37 mm, temporal duration ∼40 ms per phase, dependent on heart rate). Image analysis was performed using the cardiac analysis package of the ViewForum workstation (Philips, Best, NL). Manual tracing of the epicardial and endocardial borders was performed on the short-axis slices at end systole and end diastole by a trained radiographer. The algorithm for contour selection and subsequently calculating LV mass, systolic and diastolic parameters has been detailed elsewhere; all parameters were controlled for body surface area.^15^

### Red cell volume and PV

Red cell volume (RCV) and PV measurements were performed in the Nuclear Medicine Department of the Freeman Hospital, Newcastle upon Tyne Hospitals NHS Foundation Trust. These were calculated using the indicator dilution technique using simultaneous 51-chromium labelling of red blood cells and 125-iodine labelling of serum albumin. Radioactivity concentrations were measured in blood samples collected at 15, 30 and 45 min after reinjection and compared with standards to accurately determine the RCV and PV. Total blood volume was calculated by summing the two.^16^

Participants underwent one event each of ^51^Cr RCV (0.3 mSv effective dose) and ^125^I PV (0.06 mSv). The total effective dose for a participant completing the study was estimated at 2.76 mSv. This is equivalent to ∼13 months of exposure to background radiation in the UK.

### Statistical analysis

Continuous variables were expressed as mean±SD and comparisons made using paired t tests where groups were matched case by case and unpaired t-tests when matching was groupwise. Categorical data were expressed as proportions (%) and analysed using Fisher’s exact test. Correlation analysis was performed using parametric testing and multivariate analysis using linear regression. Analysis was performed using GraphPad Prism. Multivariate analysis was performed using SPSS.

## Results

### Cardiac MR

Cardiac MR was performed in 47 participants with CFS who were matched case by case for age and sex to 47 controls. Demographics for the two groups are shown in table 1. The mean (SD) length of history (years) for the CFS group was 14 (10) years.

**Table 1:** Cardiac magnetic resonance parameters in CFS compared with matched controls

	Controls	CFS	p Value
N	47	47	
Age (years)	47 (13)	46 (12)	NS
Females (n)	34 (72%)	34 (72%)	NS
Heart rate (/min)	64 (12)	67 (12)	NS
Systolic blood pressure (mm Hg)	124 (25)	109 (18)	**0.002**
Diastolic blood pressure (mm Hg)	76 (10)	70 (12)	**0.02**
Height (cm)	166 (26)	166 (10)	NS
Weight (kg)	73 (16)	74 (17)	NS
Ejection fraction (%)	61 (6.6)	63 (5.7)	NS
Stroke volume (mL)	73 (16.2)	57 (12.7)	**0.0002**
ED volume (mL)	121 (30.7)	91 (21.4)	**<0.0001**
ES phase (ms)	334 (44)	320 (47)	NS
ES volume (mL)	48 (18)	34 (11)	**0.0006**
ED wall mass (g)	95 (28)	70 (20)	**<0.0001**
ED wall+Pap mass (g)	102 (28)	77 (22)	**<0.0001**

Values expressed as mean (SD) normalised for body surface area unless stated.

Values in bold typeface statistically significant.

CFS, chronic fatigue syndrome; ED, end diastolic; ES, end systolic; NS, not significant.

Compared with the control group, the patients with CFS had significantly reduced end-systolic volume (ESV) and end-diastolic volume (EDV) together with reduced end-diastolic wall masses (table 1). Interestingly, both stroke volume (controlled for body surface area), systolic blood pressure (SBP) and diastolic blood pressure (DBP) were significantly lower in patients with CFS compared with controls.

### PV and RCV

A total of 41 of the patients with CFS and 10 of the controls matched groupwise went on to have assessment of plasma and RCV. Mean (SD) RCV was 1565 (443) mL with 26/41 (68%) of sufferers having values below 95% of the normal expected value for RCV. PV mean (SD) was 2659 (529) mL with 13/41 (32%) having values below 95% of the expected mean value. Total volume was lower in the CFS group compared with controls (4236 (139) vs 4396 (180)) although this did not reach statistical significance.

### Relationship with cardiac structural and functional parameters

**Figure 1:**
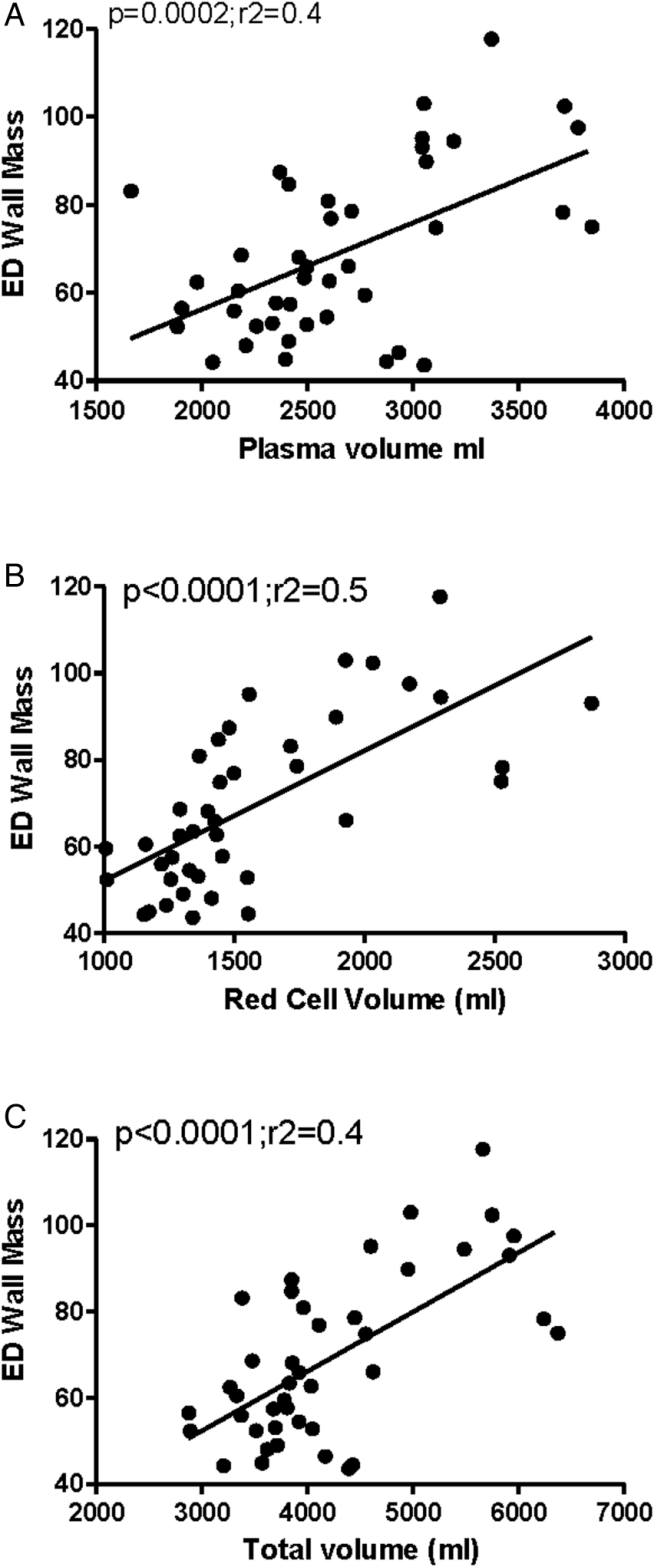
The relationship between end-diastolic (ED) wall mass and (A) plasma volume, (B) red cell volume and (C) total volume (all r=0.6).

There were strong positive correlations between total volume and end-diastolic wall mass and wall+Pap mass (figure 1A) when we considered the components of total volume increasing RCV and PV also associated with increased end-diastolic wall mass and wall+Pap mass (figure 1B, C). Interestingly, when considering the impedance cardiography, there were positive relationships between the variability of mean arterial blood pressure (MAP) and DBP (SD MAP, p=0.03, r^2^=0.1; SD DBP, p=0.02, r^2^=0.1) but not SBP and total volume. Total volume also associated negatively with cardiac index and left ventricular work index (LVWI) (p=0.004; r^2^=0.1).

### Relationships with fatigue severity and length of history

**Figure 2:**
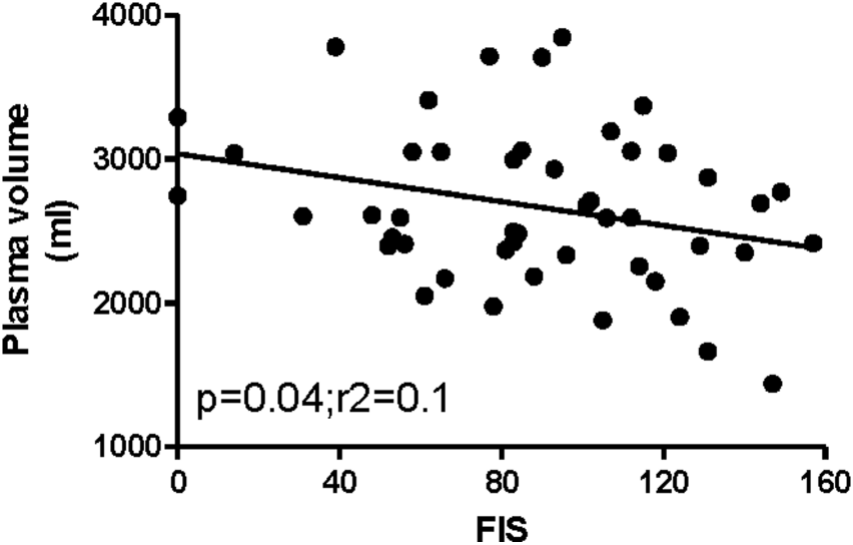
The relationship between plasma volume and fatigue measured using the Fatigue Impact Scale (FIS) (r=−0.3).

When we considered the relationship between fatigue severity, there was no relationship between increasing fatigue and total or RCV; however, there was a significant negative relationship between increasing fatigue severity measured using the FIS and lower PV (figure 2). There were no relationships between any of the MR or volume measurements and length of history suggesting that deconditioning was unlikely to be the cause of these abnormalities.

### Multivariate analysis

In order to explore the hypothesis that people with CFS have small hearts because they are physically deconditioned, we performed a linear regression with multicollinearity (no violations). We included in the model length of history and fatigue severity. End-diastolic wall mass was not associated with the length of history while controlling for factors which may be expected to determine the heart size (table 2).

**Table 2:** Linear regression multicollinearity (no violations): adjusted r^2^ 0.591, model fit: *F* 8.653, p<0.001

						95% CIs	
	B	SE	β	t	p	Lower	Upper
Constant	−3.023	31.806		−0.095	0.925	−67.98	61.934
LOH	0.079	0.239	0.04	0.33	0.744	−0.41	0.568
FIS	−0.03	0.07	−0.05	−0.427	0.672	−0.174	0.114
Age	−0.083	0.213	−0.051	−0.388	0.7	−0.517	0.351
PV%	−0.211	0.212	−0.113	−0.995	0.328	−0.644	0.222
BSA	19.852	14.18	0.222	1.4	0.172	−9.108	48.812
Sex (M)	27.123	7.147	0.614	3.795	0.001	12.528	41.718
MAP	0.347	0.181	0.227	1.917	0.065	−0.023	0.716

B, unstandardised coefficient; β, standardised coefficient.

BS, body surface area; FIS, Fatigue Impact Scale; LOH, length of history; MAP, mean arterial blood pressure; PV, plasma volume.

## Discussion

This study has confirmed, in a second, larger cohort the reduced EDVs seen in our previous studies.^3^ ^4^ Our original study has also been extended to confirm, within the same individual, the association between reduced cardiac volumes and total RCV and PV. The lack of relationship between length of disease and the MR abnormalities and PV suggests that our findings are not secondary to deconditioning. Instead, reduced cardiac volume may constitute a (pre-existing) vulnerability for developing CFS, though larger, preferably longitudinal studies would be needed to support this hypothesis. Importantly, there is also a relationship between PV and the severity of fatigue symptoms experienced by patients with CFS suggesting that this has the potential to be a therapeutic target.

Unlike the previous cardiac MR study, the current cohort was very specifically defined and excluded individuals with a formal diagnosis of depression. This therefore allows us to be definitive in our conclusion that the abnormalities detected are not secondary to the presence of depression.

The CFS cohort had significantly lower stroke index, SBP and DBP compared with the matched controls. This has been reported previously in CFS using 24 h ambulatory blood pressure measurement.^17^ This finding may represent a functional consequence of the reduced cardiac function that may explain the high prevalence of orthostatic intolerance seen in those with CFS. An alternative hypothesis is that the reduction in blood pressure is a primary problem that impacts on cardiac function as a secondary phenomenon. Either mechanism could point to a treatment target with the potential to improve quality of life in those with fatigue associated with autonomic symptoms.

In the CFS cohort, over half had RCV measurements below 95% of the expected and almost a third breached this threshold for PV. Only 10 of the control population had assessments of RCV and PV, and although there were no statistical differences between the CFS and control population, this is probably related to the limited number of controls. RCV and PV assessments have normative data available and it is interesting to determine the proportion who were below the 95% expected value which leads us to speculate, also considering that the relationship between PV and fatigue severity, that volume within the vascular system plays at least a part in the symptoms experienced by those with CFS and is a potential therapeutic target.

The direction of association between reduced PV and cardiac volumes is still unproven and further studies are needed which increase PV to determine the effect of this intervention on cardiac function and the symptoms experienced by patients with CFS. Anecdotally, patients describe symptomatic improvements with the administration of intravenous fluid.^18^ Our findings would point towards a possible explanation for this subjective improvement and future work will include interventions to restore fluid volume in patients with CFS and explore the potential amelioration of the cardiac functional impairments seen in the present study, including the progressive normalisation of LV mass. Such a study would establish the primacy of blood volume reduction and determine whether there are no primary myocardial deficits, other than those caused by low blood volume.

Our findings could provide further evidence to support the role of cardiovascular physiology as an underpinning problem in those with CFS. EDV is the volume of blood in the right and/or left ventricle at the end of load or filling in (diastole) or the amount of blood in the ventricles just before systole. As greater EDVs cause greater distention of the ventricle, EDV is often used synonymously with preload, which refers to the length of the sarcomeres in cardiac muscle prior to contraction (systole). An increase in EDV increases the preload on the heart and through the Frank-Starling mechanisms of the heart, increases the amount of blood ejected from the ventricle during systole (stroke volume). As nearly two-thirds of the blood in the systemic circulation is stored in the venous system, EDV is closely related to venous compliance. Increasing venous compliance elevates the capacitance of the veins, reducing venous return and therefore EDV. It is therefore possible that the abnormalities detected in this study represent problems arising due to impairments with venous compliance which again could potentially represent therapeutic opportunities that require further investigation.

This study has a number of limitations. It is important to recognise that although the findings represent statistical significance, whether these are clinically significant or causative needs to be further explored ideally with an appropriately designed intervention study.

This study confirms an association between reduced cardiac volumes and blood volume in CFS. The lack of relationship between length of disease and any cardiac or blood volume parameter suggests that our findings are not secondary to deconditioning. The relationship between PV and severity of fatigue symptoms does, however, suggest a potential therapeutic target in CFS.
